# Wirelessly Powered Visible Light-Emitting Implant for Surgical Guidance during Lumpectomy

**DOI:** 10.3390/s24175639

**Published:** 2024-08-30

**Authors:** Sunghoon Rho, Roy A. Stillwell, Kedi Yan, Ana Flavia Borges de Almeida Barreto, Joshua R. Smith, Patrick Fay, Alice M. Police, Thomas D. O’Sullivan

**Affiliations:** 1 Department of Electrical Engineering, University of Notre Dame, Notre Dame, IN 46556, USA; 2 Department of Electrical and Computer Engineering, University of Washington, Seattle, WA 98195, USA; 3 Allen School of Computer Science and Engineering, University of Washington, Seattle, WA 98195, USA; 4 Monument Health Cancer Care Institute, 353 Fairmont Boulevard Rapid City, Rapid City, SD 57701, USA

**Keywords:** implantable device, wireless power transfer, surgical guidance, breast cancer, localization

## Abstract

Achieving negative surgical margins, defined as no tumor found on the edges of the resected tissue, during lumpectomy for breast cancer is critical for mitigating the risk of local recurrence. To identify nonpalpable tumors that cannot be felt, pre-operative placements of wire and wire-free localization devices are typically employed. Wire-free localization approaches have significant practical advantages over wired techniques. In this study, we introduce an innovative localization system comprising a light-emitting diode (LED)-based implantable device and handheld system. The device, which is needle injectable and wire free, utilizes multiple wirelessly powered LEDs to provide direct visual guidance for lumpectomy. Two distinct colors, red and blue, provide a clear indication of tissue depth: blue light is absorbed strongly in tissue, visible within a close range of <1 cm, while red light remains visible through several centimeters of tissue. The LEDs, integrated with an impedance-matching circuit and receiver coil, are encapsulated in biocompatible epoxy for injection with a 12 G needle. Our findings demonstrate that the implant exhibits clearly perceivable depth-dependent color changes and remains visible through >2 cm of ex vivo chicken breast and bovine muscle tissue using less than 4 W of transmitted power from a handheld antenna. These miniaturized needle-injectable localization devices show promise for improving surgical guidance of nonpalpable breast tumors.

## 1. Introduction

Over 287,000 lumpectomy procedures are performed each year in the US, of which nonpalpable breast lesions that cannot be felt account for at least 60% [1,2,3,4]. Surgical removal of these nonpalpable lesions requires the use of localization techniques to guide the surgeon during a lumpectomy. One technique in use today is wire localization (WL), which entails the pre-operative implantation of ~7 cm long wires under imaging guidance to bracket the lesion location [5]. The implanted distal end of the wire contains a tissue anchor, while the proximal end of the wire protrudes from the skin. The surgeon can identify the tumor’s location following the direction of the wire and through palpation of the wire [6].

However, the WL technique presents several practical and logistical hurdles. First, the procedure is uncomfortable for patients since the wire must be placed on the day of surgery, typically in coordination with the radiology department, and patient movement is limited once placed. Moreover, WL complications can include displacement or migration of the wire in the time before surgery as well as retention of wire fragments [7,8].

To overcome this limitation, several “wire-free” localization devices have been recently developed based on different modalities: radioactive, magnetic, and radio-frequency [9]. These approaches disassociate the breast localization procedure from the surgical schedule and provide greater flexibility and comfort for patients. Though wire-free localization does not result in fewer positive margins or a reduced rate of re-excision, it does result in significant cost savings compared to WL [10]. Current wire-free localization methods involve the pre-operative image-guided placement of millimeter-sized implants (size range 1–2 mm by 5–12 mm) that can be noninvasively detected utilizing an external handheld sensing probe as summarized in Table 1 [11,12,13,14].

Even though wire-free localization devices are rapidly becoming popular for tumor localization, there are some drawbacks. Most are not well-suited for differentiating multiple lesions in close proximity (~2 cm—minimum distance to adjacent marker) due to overlapping signals [13,15]. Further, most localization units typically provide only a single dimension of feedback (e.g., distance to the implant), which can make 3D localization difficult to visualize. Finally, most of the magnetic wire-free localization devices are unsuitable for use during breast MRI because they introduce imaging artifacts [11,13,16,17].

**Figure 1:**
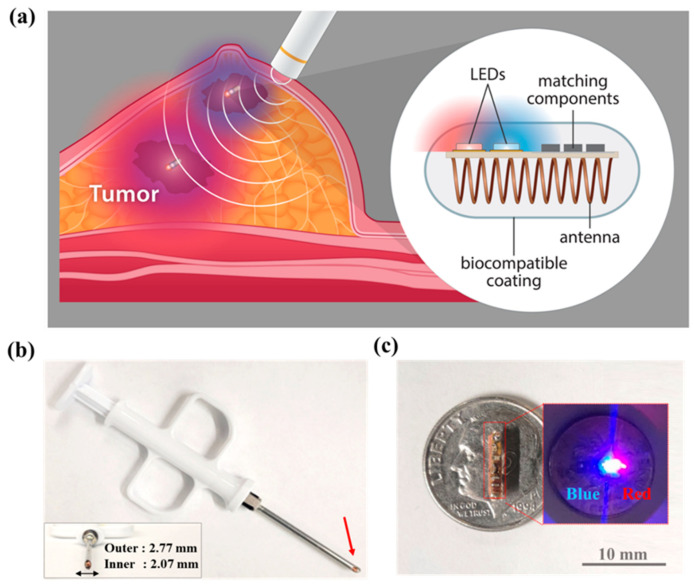
Illustration of the principle of the dual-color wire-free localization system is shown in (**a**). Different tissue attenuation of red (far) and blue (close) light indicates distance to the nonpalpable lesion. (**b**) shows the localization device loaded into a tissue introducer tool (12 G) at the red arrow. An image of the wirelessly powered device with light emission is shown in (**c**).

In this study, we present a needle-injected, wirelessly powered visible light-emitting localization device to overcome the drawbacks of WL and existing wire-free localization approaches. An illustration describing the use of the proposed device is shown in Figure 1a. The device is designed to fit in a 12 gauge (2.16 mm inner diameter) biopsy needle so that it can be introduced during a standard breast biopsy procedure (Figure 1b). The device includes red (624 nm) and blue (465 nm) LEDs that are activated via wireless power transfer (WPT) from an external antenna (Figure 1c). Due to significantly different tissue optical attenuation (red vs. blue), a novel feature of the device is that it can provide the surgeon direct visible guidance to help judge the direction and distance to the lesion. Additionally, it is possible that the device could also be used to localize multiple lesions in close proximity.

In this paper, we describe the implant design, which consists of multiple-colored LEDs in anti-parallel configuration, a receiver coil, and impedance-matching circuitry with a biocompatible epoxy. The circuit was designed to minimize implantable device size (volume 27 mm^3^, 2 × 9 mm cylinder) using off-the-shelf components and a unique anti-parallel LED configuration for rectification that reduces the number of required components [18,19]. The device is designed to operate in the 6.78 MHz industrial, scientific, and medical (ISM) RF band, which is frequently used for biomedical applications and causes limited tissue absorption and heating [20]. On the transmitting side, a handheld 5.5 cm × 5.5 cm × 0.6 cm prototype antenna was designed for wireless power transfer (WPT) activation of the implant. We found that the localization device is visible through 3 cm depth of ex vivo chicken breast. Furthermore, the color changes at the different depths are clear, showing a red light in the 3 cm depth transitioning to a purple/blue color at the 0.5 cm depth in the chicken breast. In addition, we report that the device works in higher optically attenuating bovine muscle tissue at a 2 cm depth, with the color transitioning from red to pinkish as it is close to the surface of the tissue using a 4 W WPT transmitter (TX). Our results indicate that the device and handheld probing system has the potential to enhance existing wire-free localization devices for surgical guidance of nonpalpable breast lesions. To the best of our knowledge, this is the first presentation of a visible light-emitting localization device for cancer surgery.

## 2. Materials and Methods

### 2.1. Overview of the Wirelessly-Powered LED-Based Localization Device

The localization device is designed to provide a visual color-based indication of its tissue position and depth based on differences in tissue optical attenuation at different wavelengths. For instance, the approximate absorption coefficients (*μ*_*a*_) for blue light (460 nm) in adipose and malignant breast tissue are 1.5 cm^−1^ and 2.6 cm^−1^, showing that the blue is expected to be absorbed in less than a centimeter of tissue [21,22,23,24]. In contrast, red (650 nm) is visible up to several centimeters deep due to its lower tissue absorption: *μ*_*a*_= 0.08 cm^−1^ (adipose) and *μ*_*a*_ = 0.11 cm^−1^ (malignant) [23,25,26,27,28,29]. We chose to use miniature 0402 size (1.0 × 0.5 mm) surface mount LEDs: a red (624 nm APG1005SECET, Kingbright, New Taipei City, Taiwan) and blue (465 nm 150040BS73220, Würth Elektronik, Niedernhall, Germany) in the prototype implant.

### 2.2. Device Receiver Antenna Design

In order to be compatible with standard breast biopsy procedures, the localization device must fit into a biopsy needle diameter of 12 gauge or smaller [30,31]. This poses a size and shape restriction on the antenna. The antenna also needs to resonate at the desired frequency of 6.78 MHz, which was chosen due to its low tissue attenuation that results in negligible tissue heating at low RF power. For example, powers of up to 25 W have been used in a similar antenna design resulting in a specific absorption rate (SAR) well below safety limits [32].

**Figure 2:**
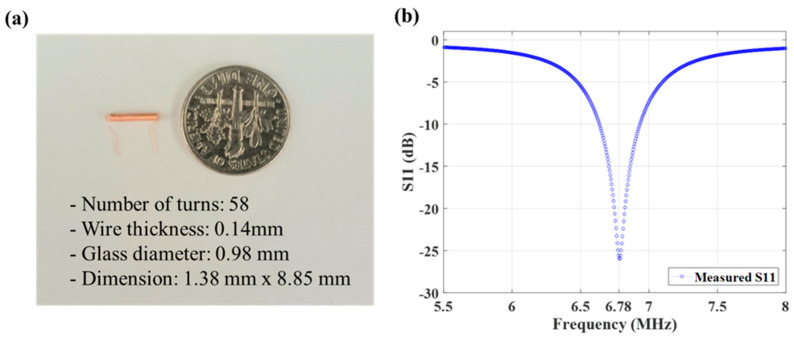
Characterization of the fabricated receiver antenna. (**a**) The receiver antenna with 58 turns. The measured return loss at the intended frequency (6.78MHz) is presented in (**b**).

Considering the limited options, we chose to wind a coil into a helix, resulting in a normal mode helical antenna that radiates its signal perpendicularly to the axis of the helix. This design allows the antenna to be shorter in length compared to a standard dipole antenna at the same frequency, while having a cylindrical shape compatible with needle injection. This makes the helical antenna well suited for our application, combining compactness, efficiency, and ease of integration [33,34,35]. Thus, the receiver (RX) coil dimension consists of a 36 gauge copper wire wound around a 1.0 mm diameter glass cylinder with 58 turns (Figure 2a) and measures 1.38 mm × 8.85 mm. The number of turns and the size of the glass diameter were deliberately chosen to fit within a 12 gauge and to achieve a TX-RX separation of up to 3 cm.

Though a ferrite core could improve WPT efficiency, a nonmagnetic core was chosen to minimize MRI artifacts [36]. This antenna design (1.38 mm diameter, 58 turns) leads to a resonant impedance match to a 50 ohm load at the intended frequency (S_11_ return loss of −26 dB at 6.78 MHz, Figure 2b).

### 2.3. WPT Circuit Design

**Figure 3:**
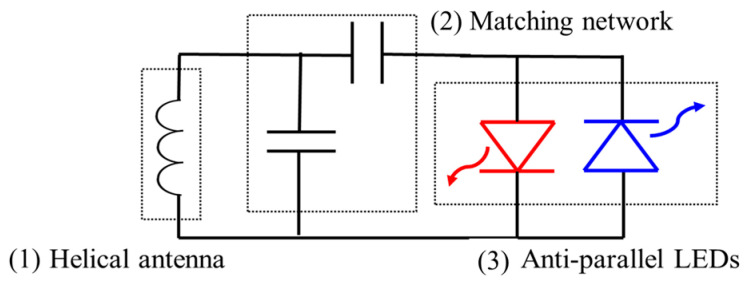
The circuit for the designed implant is presented. (1) Helical antenna for the wireless power transfer, (2) matching network, and (3) antiparallel pair of LEDs.

The localization device includes a simple circuit to rectify the incoming power and maximize the wireless power delivery to the LED load. Due to the small size constraints, we minimized the number of components required at the expense of WPT efficiency based on our previous work [18,19]. Specifically, the LEDs themselves serve as a rectifier in an anti-parallel configuration and an L topology matching network was designed and shown in Figure 3 [37].

**Figure 4:**
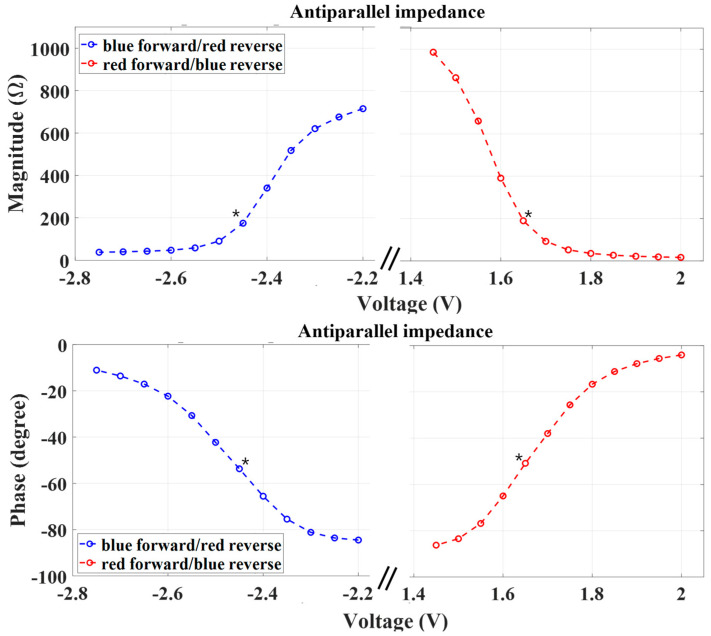
The measured impedance of the anti-parallel pair LEDs: magnitude (**top**) and phase (**bottom**). * Marks present the targeted impedance of the load in forward/reverse bias.

A Pi or T-section matching network topology could also be used to improve WPT efficiency, but it was not chosen since the additional components (especially an inductor) will increase the overall implant size. Since the LEDs have a nonlinear impedance that varies with the diode bias and the IV characteristics of the blue and red LEDs are different, we used an iterative empirical approach to optimize the matching network. We first characterized the individual impedance of LEDs at both forward and reverse bias since the LEDs are connected in an antiparallel configuration as shown in Figure 3. Then, the combined impedance of blue forward/red reverse and red forward/blue reverse was calculated to estimate the optimal load impedance, as shown in Figure 4.

To design the matching network, we hypothesized that the average impedance at the desired operating voltages (i.e., red forward/blue reverse and blue forward/red reverse) would provide the best estimation of the load impedance for optimal operation [18,19]. For instance, we chose a target bias voltage (impedance) of 1.65 V (120–147.2j Ω) for the red forward/blue reverse LED and 2.45 V (103.4–140.7j Ω) for the blue forward/red reverse LED based on the threshold voltage of the LEDs (marked with a *). Then, the averaged impedance was chosen as the effective load impedance and a matching network was designed to match this load to the impedance of the RX antenna (1.06 + 19.28j Ω) at 6.78 MHz.

The L-section matching network at 6.78 MHz for these impedances can be achieved with the capacitor-capacitor (CC) L-network (1117 pF shunt and 1189 pF series capacitors). However, because of the simplicity of our impedance estimation approach and the parasitic effects of the printed circuit board (PCB) layout in the implant, the optimal impedance varies slightly [38]. Therefore, we empirically and iteratively investigated several shunt and series capacitances beginning with the initial values calculated as described. We found that a parallel shunt combination of a 1100 pF (5% tolerance) capacitor and an 82 pF (5% tolerance) capacitor, along with a 1200 pF (5% tolerance) series capacitor, allowed for sufficient WPT to achieve visible implant operation at a TX-RX distance separation of up to 3 cm in the air.

### 2.4. Wireless Powering System

**Figure 5:**
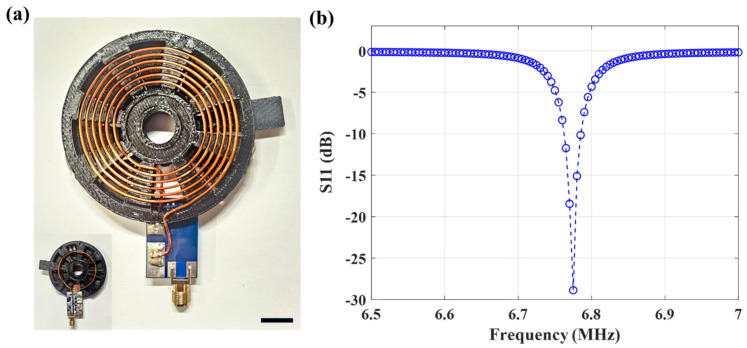
Characterization of the fabricated handheld transmitting antenna. (**a**) Photograph of the transmitting antenna front and back (inset). (**b**) The measured return loss at the 6.78 MHz operating frequency. Scale bar = 10 mm.

WPT was driven by a continuous wave (CW) RF source operating at 6.78 MHz (Wibotic TR-301). The controller manages the power output of the WPT, ranging from 0 W to 250 W. For device evaluation, 2, 3, and 4 W of CW transmitting power was utilized [39]. A handheld TX antenna using loop-coils and measuring 5.5 cm × 5.5 cm × 0.6 cm was designed and is shown in Figure 5a. The TX antenna resonates at the intended frequency (6.78 MHz) with an S_11_ return loss of −29 dB as shown in Figure 5b.

### 2.5. Device Optical Power Evaluation

Due to factors such as impedance matching network efficiency, parasitic resistance from the PCB, and efficiency of the LEDs, we chose to directly measure optical output from the wirelessly powered device to characterize overall system power transfer efficiency. Furthermore, optical power is an important practical characteristic of our device for localization and sufficient visibility. To assess optical power output, the device was directly attached to an external silicon photodiode (FDS1010, Thorlabs Inc., Newton, NJ USA) using tape and powered wirelessly. The diagram of the setup for measuring the optical power appears in a previously published paper [19]. Separate measurements of optical output power of the red and blue LEDs were performed by covering one with opaque black tape. The photodiode was connected to an ammeter, and reverse biased at 1 V. The measured photocurrent was converted to power, given the photodiode’s responsivity. To mitigate the effects of ambient light, the laboratory lights were turned off during the measurement and dark current was subtracted from the measured value. The distance from the wireless TX to the device was controlled using linear translational stages. A 3D-printed mount was created to securely hold the photodiode on the linear stage.

### 2.6. Device Temperature Measurement

**Figure 6:**
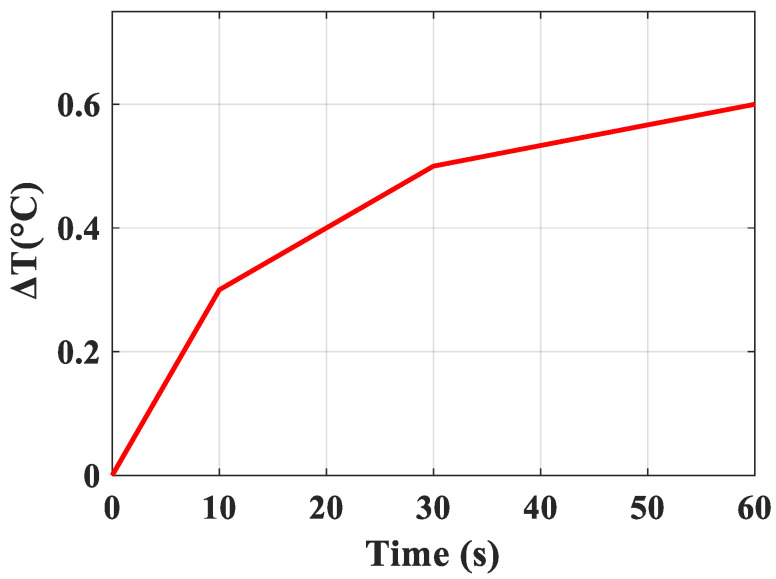
Heat generation by LEDs under RF power at 4 W in air.

Since excessive heat generated by the LEDs can cause cell death and tissue damage, we measured the device’s temperature changes over time in the air by using an infrared thermal camera (FLIR ONE Camera, Teledyne FLIR, Wilsonville, OR, USA). The device starts at 26.2 °C (i.e., room temperature) and begins to saturate at 26.8 °C after 60 s as shown in Figure 6. The temperature change over this time is less than 1.0 °C, which is relatively small and safe for tissue, minimizing the risk of damage in the breast [40]. Furthermore, in this surgical localization application, any locally heated tissue will be immediately excised anyway.

## 3. Results

### 3.1. Measurement of Optical Power Emission

**Figure 7:**
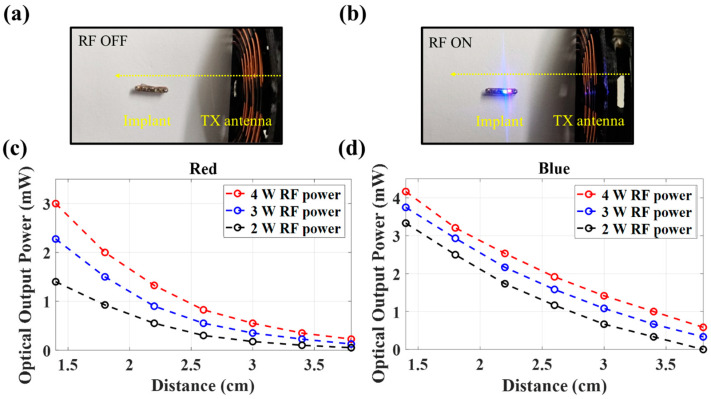
The designed implant is shown with RF OFF (**a**) and ON (**b**). Measured optical output of the LEDs as a function of the distance to the transmitter antenna is shown for the (**c**) red and (**d**) blue LEDs.

The implant is shown in Figure 7, with RF off (a) and on (b). The optical output from the implant was measured as a function of the RX/TX separation through the air at a RF power of 2, 3, and 4 W using a photodiode (Figure 7c,d). Light output from the implant is measurable at up to approximately 4 cm in TX-RX distance. With 4 W RF power, the red LED emits over 0.5 mW at distances up to 3 cm. The measured optical output power of blue LED also exceeds 0.5 mW up to 3.0 cm at all tested RF power levels.

The overall power transmission efficiency (optical output power relative wireless transmission power) ranges from approximately 0.2% to 0.5%, which is consistent with other devices of similar size [41,42].

**Figure 8:**
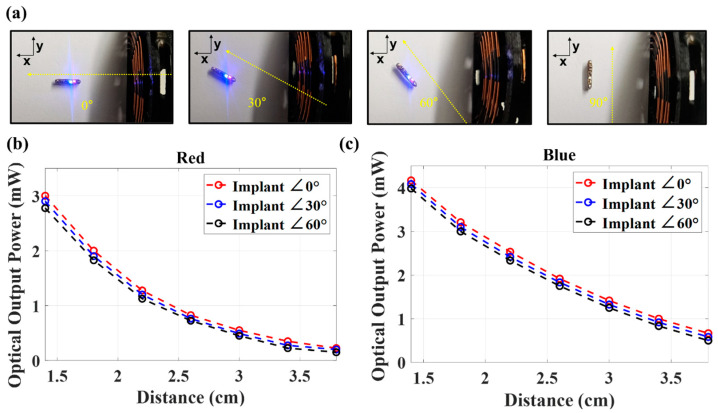
Implant activation at different angles with respect to the transmitting antenna are presented in (**a**). Measured optical output of the LEDs as a function of the distance of the matched impedance circuit at different angles for the (**b**) red and (**c**) blue LEDs.

Depending upon tissue placement, the implant’s orientation relative to the TX antenna can vary. We therefore evaluated how WPT efficiency varies as a function of the angle between the localization implant and TX antenna as shown in Figure 8a. Using 4 W transmit power, the optical output was measured at 0°, 30°, 60°, and 90°. The emission power was similar at angles up to 60°, demonstrating a wide-angle acceptance, before decreasing and becoming immeasurable at 90° as shown in Figure 8b (red) and Figure 8c (blue). Overall, the results shown in Figure 8 indicate that the device can be utilized as a localization device across wide angles.

**Figure 9:**
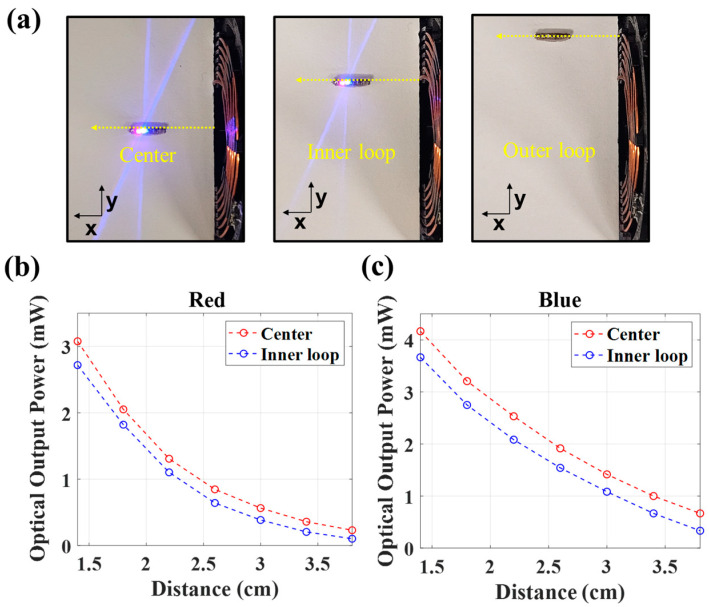
Effect of lateral misalignment configuration of the TX antenna and localization device on optical power output. Implant activation at three different alignments corresponding to the center, inner loop, and outer loop of the TX antenna is presented in (**a**). The optical output power for red and blue at the center and inner loop is indicated in (**b**,**c**).

The effect of lateral misalignment of the TX-RX on WPT is also an important factor to assess for our localization system since surgeons will manually position the handheld antenna to locate the device. Therefore, we measured the optical output power at three points of lateral alignment corresponding to the center, the inner loop, and the outer loop of the TX antenna. The measurements show that there is little loss in optical power from the center position to the inner loop, as shown in Figure 9b,c, which is 14 mm away. However, when positioned at the outer loop (25 mm from the center), the device shuts off, as shown in Figure 9a. Therefore, given that the inner diameter of the TX antenna is 28 mm, these results indicate that the handheld antenna must be oriented laterally within about 3 cm.

### 3.2. Ex Vivo Tissue Demonstration and Evaluation of the Localization System

To demonstrate the localization approach in tissue, we tested the system using two different tissues that recapitulate the range of optical properties found in normal human breast and breast tumors: chicken breast and bovine muscle tissue. Per the literature, the optical absorption of chicken breast is approximately 0.1 cm⁻^1^ at 650 nm and 0.7 cm⁻^1^ at 460 nm [43]. It is expected that the red absorption will be similar in human breast tissue (0.08–0.11 cm^−1^ range of human breast adipose and tumor tissue), but the blue absorption will be much higher in human tissue (1.5–2.6 cm^−1^ range). Since chicken breast has a lower optical scattering than human breast tissue, bovine muscle tissue was also used. Bovine muscle tissue has approximately 10 times higher absorption at 650 nm (1.0 cm⁻^1^) and 3–5 times higher absorption at 460 nm (8.0 cm⁻^1^) than human breast tissue. Though the bovine muscle tissue has much more overall attenuation than human tissue, successful operation in this tissue provides confidence that the device would work in high vascularized tumors [44]. For durability and biocompatibility, we coated the localization devices with a biocompatible clear epoxy (MED-301, Epoxy Technology Inc., Billerica, MA, USA).

**Figure 10:**
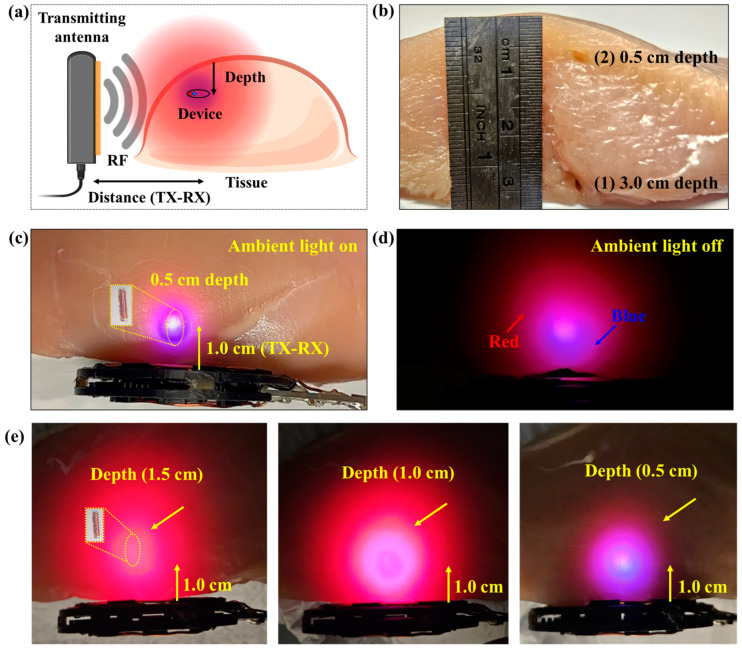
Illustration of ex vivo tissue demonstration experiments. (**a**) Schematic view of ex vivo localization experiment showing the relative position of the transmitting antenna and the implantable device in the tissue. (**b**) The measured implant depth in the tissue, where the holes indicate the location of the device. Demonstration of close-range illumination where the implant is 0.5 cm deep with ambient light on (**c**) and off (**d**). Depth evaluation for localization of the implant at 0.5, 1.0, and 2.5 cm is shown in (**e**). (The exposure time of the camera was adjusted down to avoid the saturating the camera sensor and faithfully reproduce what is seen with the naked eye.)

The device was placed at different depths (0.5 cm and 3.0 cm) into the chicken using a 12 gauge introducer, with the TX-RX distance as shown in Figure 10a,b. At the 0.5 cm depth, the individual red and blue colors from the implant are observed, as well as purple mixing, as shown in Figure 10c. Even with the ambient light on, at this shallow depth, the optical power from the device is significant enough to visualize the location of the implant using the two colors. The light is much more obvious when the ambient light is off, as shown in Figure 10d. Moreover, the blue color becomes much more visible as the implant depth is reduced from 1.5 cm to 0.5 cm (Figure 10e), indicating that it could be utilized for precise localization, as shown in Figure 10e. These experiments demonstrate that the device creates a distinctive pattern of red and blue colors at shallow depths in chicken breast tissue.

**Figure 11:**
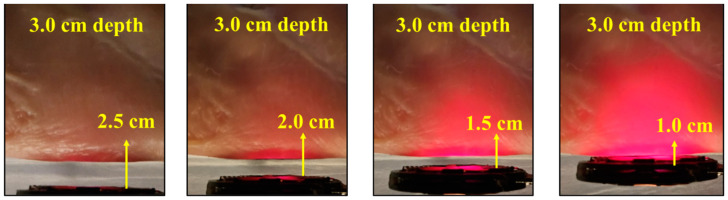
Demonstration of deep tissue localization. Light emission of the device shown for an implant depth of 3 cm at transmitter-receiver separations ranging from 1.0 to 2.5 cm.

To evaluate the deeper localization at 3.0 cm depth, we placed the device in the position shown in Figure 10b. Then, the TX antenna was placed at lateral distances 2.5 cm, 2.0 cm, 1.5 cm, and 1.0 cm away from the implant to test the visibility of the light. As expected, due to optical attenuation, the light only shows the red color at the 3.0 cm depth, as shown in Figure 11. In addition, when the TX antenna is close to the device, the light is much more obvious since the light emitting power is increased as shown in Figure 11. This result is consistent with optical attenuation in tissue: red light is attenuated less, and blue light is attenuated more. This evaluation proves that the device can be utilized for locating deeper positions, indicated by the red light only. Overall, Figure 10 and Figure 11 demonstrate that the device can provide visual guidance for localization through color changes.

Several videos are included online as Supplementary Information that show the operation of the optically-enhanced localization system in real-time with implants placed at 0.5, 1.5, and 3.0 cm deep in ex vivo chicken breast tissue.

**Figure 12:**
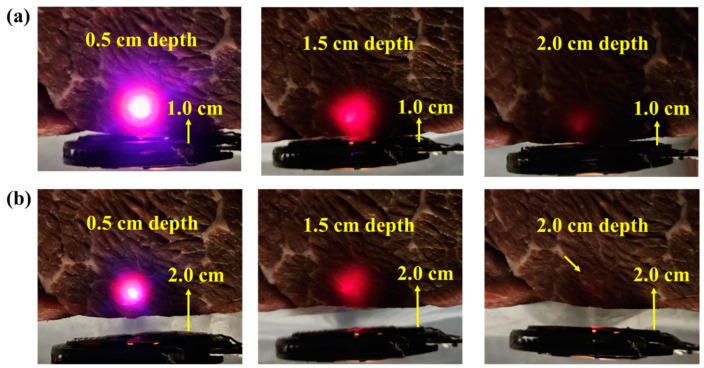
Demonstration of visible wire-free localization implant in bovine muscle tissue with room lights off. The transmitter-receiver distance is fixed at 1.0 cm (**a**) and 2.0 cm (**b**) for implant depths of 0.5, 1.5, and 2.0 cm.

Furthermore, we tested the device in bovine muscle tissue for localization. This is because the optical properties of chicken breast at shorter wavelengths (i.e., the blue LED) differ from those of human tissue due to the presence of hemoglobin in humans [23,43]. Therefore, we tested our device in bovine muscle tissue to estimate the color changes in the presence of significant amounts of hemoglobin. The device was injected and placed at depths of 0.5 cm, 1.5 cm, and 2.0 cm from the surface, with the TX antenna located 1.0 cm away from the device, as shown in Figure 12a. As expected, a pinkish color, which is a combination of red and blue, appears when the device is close to the surface. In contrast, only the red color appears at depths of 1.5 cm and 2.0 cm. Moreover, the device operates at up to a 2 cm TX-RX separation, as shown in Figure 12b. These results consistently demonstrate that our device can be utilized for localization in the presence of higher optical attenuation and can still service as an indicator of depth.

## 4. Discussion

Over the past decade, breast surgeons have been transitioning away from the use of WL in favor of newer wire-free approaches because WL has presented clear disadvantages for patients and physicians for localizing nonpalpable breast lesions during lumpectomy [45,46]. In addition to a risk for wire migration or displacement [47,48], WL requires scheduling of wire placement on the same day of surgical excision, the coordination of which can cause challenges in scheduling and delayed surgical start times [49]. Wire-free localization has been shown to be an effective alternative for the localization of nonpalpable breast lesions, can be implanted at least 30 days before surgery, has shown no adverse effects, and has a lower cost [10,50,51].

However, existing wire-free localization approaches have limitations. Current devices cannot be separately identified when placed closer than 18–20 mm [52], which hampers the localization of the multiple lesions in close proximity. Furthermore, wire-free localization units usually only provide feedback in a single dimension (such as distance to the implant) [53], which makes it difficult to visualize in 3D [46].

To address these limitations, we designed and demonstrated a 12 gauge biopsy needle-injectable, optically enhanced device intended for intraoperative surgical localization of nonpalpable breast lesions. This device is the first wireless optical-based localization device designed for lumpectomy surgeries. Utilizing an anti-parallel configuration of LEDs and when powered by an external wireless power source, it emits light in a miniature needle-injectable format [19]. The device emits perceptible visible light using only a few watts of RF transmission power. The light from the device is visible from up to 3 cm deep in ex vivo chicken breast tissue and 2 cm in bovine muscle tissue. These results are promising, as one study shows that 153 out of 154 localization devices for breast tumors were placed at a mean depth of 2.6 cm from the skin surface and a mean distance of 2.8 mm from the lesions when the patient was positioned supine during surgery [54].

To demonstrate future translation of the localization system to a real clinical surgical environment, a transmitting probe was designed with a size of 5.5 × 5.5 × 0.6 cm and was easily manipulated by hand. We also found that the implantable device has a wide angular range of operation measured at up to 60°. Moreover, the device design could be attractive for other biomedical applications, such as optogenetic stimulation, photobiomodulation, and photoactivated therapy [19,55]. For instance, our previous study shows that a wirelessly powered device based upon a similar design used in this study triggers pyroptosis in colorectal cancer cells [19].

Our study of the optically enhanced localization approach has the following limitations. First, the device requires a relatively large 12 gauge biopsy needle. In comparison, other implantable devices for localization can be administered using needles ranging from 16 G to 18 G [13]. In this case, we wound the copper wire around a 1 mm diameter glass core, which is small enough to fit into a 12 gauge biopsy needle when soldered to the PCB board. This antenna currently limits the minimum size of the device to the inner diameter of 12 gauge biopsy needle. One approach we can apply for future development is to reposition the coils to the outer surface of the device. By implementing outer surface coiling, we can increase the antenna’s inductance, which can improve power delivery.

Second, we tested our device using ex vivo chicken breast and bovine muscle tissue; however, both tissues have different absorption spectra to fresh human tissue [23,43,44,56]. Specifically, the optical absorption of the chicken breast at the red 650 nm wavelength is similar to human; however, the blue will absorb less [43]. As a result, we expect more visual contrast and depth selectivity based on the two colors in human tissue. The bovine muscle tissue tested has significantly higher absorption at both wavelengths compared to human tissue [23,44,56,57]. Thus, we expect the blue/red light emitted by the device will be more visible in human tissue. Third, we also only tested the device in the laboratory environment, and not in the presence of the bright surgical lights used in an operating theater, which is likely to reduce contrast significantly. However, it is possible for surgical lights to be dimmed or deactivated temporarily during the localization procedure. Finally, the MRI safety of the metal-containing device still needs to be evaluated, even though it does not have any magnetic components.

Future work includes testing the optical guidance system within fresh mastectomy specimens, which exhibit more realistic optical absorption due to the presence of hemoglobin components [22,58], and in an operating theatre with bright lighting. In addition, an enclosed transmitting probe will be developed, making it practical for use in the operating theater. Finally, the potential of our device for improving the localization of multiple lesions will be tested.

## 5. Conclusions

We report the design and ex vivo testing of a wirelessly powered visible light-emitting implant for surgical guidance during lumpectomy with two LEDs. The cylindrical needle-injectable device measures 2 × 9 mm with a volume of 27 mm^3^ and is small enough to fit into a 12 gauge biopsy needle. The biocompatible prototype emits up to several milliwatts of optical power when excited with only 4 W of wireless RF power using a handheld probe. The circuit employs antiparallel LEDs to serve as a rectifier, which minimizes the number of required components and thus overall size. When implanted in ex vivo chicken breast and bovine muscle tissue, the implant is activated and visible at depths up to 2 cm. Due to tissue optical attenuation, the perceived color of light emission through the tissue demonstrates depth of the implant such that blue/purple light indicates shallow depth while red light indicates increased depth. This technology has the potential to enhance current wire-free localization devices by providing the surgeon with direct visual guidance.

## Figures and Tables

**Table 1:** Comparison of features of various wire-free localization systems [13].

	Radioactive Seed	Radar Reflector	Magnetic Seed	Radiofrequency ID	Visible Light Localization (This Work)
Manufacturer (brand name)	IsoAid	Merit Medical (Scout)	Stryker (MOLLI)	Hologic (LOCalizer)	N/A
Maximum depth for detection	None	6 cm	4 cm	3~6 cm	3~4 cm
Introducer gauge	18 gauge	16 gauge	18 gauge	12 gauge	12 gauge
Size implanted device	4.5 mm × 1 mm	12 mm × 1.6 mm	5 mm × 1 mm	11 mm × 2 mm	9 mm × 2 mm
Minimum distance to adjacent marker	2 cm	2 cm	2 cm	2 cm	0 cm
Detection technology	Gamma detector	Infrared trigger, electromagnetic Signal	Alternating magnetic field induces magnet in seed	RFID trigger and signal	Visible light

## Data Availability

Data are contained within the article.

## Supplementary Materials

The following supporting information can be downloaded at: https://www.mdpi.com/article/10.3390/s24175639/s1.

## References

[1] Hall M.J., Schwartzman A., Zhang J., Liu X. (2017). Ambulatory Surgery Data from Hospitals and Ambulatory Surgery Centers: United States, 2010. https://europepmc.org/article/med/28256998.

[2] Lovrics P., Cornacchi S., Vora R., Goldsmith C., Kahnamoui K. (2011). Systematic review of radioguided surgery for non-palpable breast cancer. Eur. J. Surg. Oncol. (EJSO).

[3] Giaquinto A.N., Miller K.D., Tossas K.Y., Winn R.A., Jemal A., Siegel R.L. (2022). Cancer statistics for African American/Black People 2022. CA Cancer J. Clin..

[4] Fosko N.K., Gribkova Y., Krupa K., Jain K., Moore D., Chen C., Potdevin L., Kumar S., Eladoumikdachi F., Kowzun M.J. (2023). The use of intraoperative ultrasound during breast conserving surgery. Clin. Breast Cancer.

[5] Hayes M.K. (2017). Update on preoperative breast localization. Radiol. Clin. N. Am..

[6] Snider H.C., Morrison D.G. (1999). Intraoperative ultrasound localization of nonpalpable breast lesions. Ann. Surg. Oncol..

[7] Yim J.H., Barton P., Weber B., Radford D., Levy J., Monsees B., Flanagan F., Norton J.A., Doherty G.M. (1996). Mammographically detected breast cancer. Benefits of stereotactic core versus wire localization biopsy. Ann. Surg..

[8] Montrey J.S., Levy J., Brenner R. (1996). Wire fragments after needle localization. AJR Am. J. Roentgenol..

[9] Mayo R.C., Kalambo M.J., Parikh J.R. (2019). Preoperative localization of breast lesions: Current techniques. Clin. Imaging.

[10] Nguyen C.L., Cui R., Zhou M., Ali F., Easwaralingam N., Chan B., Graham S., Azimi F., Mak C., Warrier S. (2024). Cost-Effectiveness of Radar Localisation Versus Wire Localisation for Wide Local Excision of Non-palpable Breast Cancer. Ann. Surg. Oncol..

[11] Mango V., Ha R., Gomberawalla A., Wynn R., Feldman S. (2016). Evaluation of the SAVI SCOUT surgical guidance system for localization and excision of nonpalpable breast lesions: A feasibility study. Am. J. Roentgenol..

[12] Garzotto F., Comoretto R.I., Michieletto S., Franzoso G., Mele M.L., Gregori D., Bonavina M.G., Bozza F., Caumo F., Saibene T. (2021). Preoperative non-palpable breast lesion localization, innovative techniques and clinical outcomes in surgical practice: A systematic review and meta-analysis. Breast.

[13] Kasales C. (2022). Wireless localization of breast lesions: An update. Semin. Roentgenol..

[14] Lowes S., El Tahir S., Koo S., Amonkar S., Leaver A., Milligan R. (2023). Pre-operative localisation of axillary lymph nodes using radiofrequency identification (RFID) tags: A feasibility assessment in 75 cases. Clin. Radiol..

[15] Jadeja P.H., Mango V., Patel S., Friedlander L., Desperito E., Ayala-Bustamante E., Wynn R., Chen-Seetoo M., Taback B., Feldman S. (2018). Utilization of multiple SAVI SCOUT surgical guidance system reflectors in the same breast: A single-institution feasibility study. Breast J..

[16] Mango V.L., Wynn R.T., Feldman S., Friedlander L., Desperito E., Patel S.N., Gomberawalla A., Ha R. (2017). Beyond wires and seeds: Reflector-guided breast lesion localization and excision. Radiology.

[17] Harvey J.R., Lim Y., Murphy J., Howe M., Morris J., Goyal A., Maxwell A.J. (2018). Safety and feasibility of breast lesion localization using magnetic seeds (Magseed): A multi-centre, open-label cohort study. Breast Cancer Res. Treat..

[18] Rho S., Stillwell R.A., Fay P., Ludwig K.K., O’Sullivan T.D. (2022). Optically-enhanced wireless breast lesion localization device for use during lumpectomy. Advanced Biomedical and Clinical Diagnostic and Surgical Guidance Systems XX.

[19] Rho S., Sanders H.S., Smith B.D., O’Sullivan T.D. (2024). Miniature wireless LED-device for photodynamic-induced cell pyroptosis. Photodiagn. Photodyn. Ther..

[20] Karimi M., Jouaicha H., Lellouche F., Bouchard P.-A., Sawan M., Gosselin B. A 6.78-MHz robust WPT system with inductive link bandwidth extended for cm-sized implantable medical devices. Proceedings of the 2020 42nd Annual International Conference of the IEEE Engineering in Medicine & Biology Society (EMBC).

[21] Tromberg B.J., Zhang Z., Leproux A., O’Sullivan T.D., Cerussi A.E., Carpenter P.M., Mehta R.S., Roblyer D., Yang W., Paulsen K.D. (2016). Predicting Responses to Neoadjuvant Chemotherapy in Breast Cancer: ACRIN 6691 Trial of Diffuse Optical Spectroscopic ImagingOptical Imaging of Breast Cancer Chemotherapy Response. Cancer Res..

[22] O’Sullivan T.D., Cerussi A.E., Tromberg B.J., Cuccia D.J. (2012). Diffuse optical imaging using spatially and temporally modulated light. J. Biomed. Opt..

[23] Ghosh N., Mohanty S., Majumder S., Gupta P. (2001). Measurement of optical transport properties of normal and malignant human breast tissue. Appl. Opt..

[24] Palmer G.M., Zhu C., Breslin T.M., Xu F., Gilchrist K.W., Ramanujam N. (2006). Monte Carlo-based inverse model for calculating tissue optical properties. Part II: Application to breast cancer diagnosis. Appl. Opt..

[25] Vasudevan S., Forghani F., Campbell C., Bedford S., O’Sullivan T.D. (2020). Method for quantitative broadband diffuse optical spectroscopy of tumor-like inclusions. Appl. Sci..

[26] Vasudevan S., Campbell C., Liu F., O’Sullivan T.D. (2021). Broadband diffuse optical spectroscopy of absolute methemoglobin concentration can distinguish benign and malignant breast lesions. J. Biomed. Opt..

[27] O’Sullivan T.D. (2021). Diffuse optical spectroscopy from bench to bedside to wearable to implant. Multiscale Imaging and Spectroscopy II.

[28] Stillwell R.A., Kitsmiller V.J., Wei A.Y., Chong A., Senn L., O’Sullivan T.D. (2021). A scalable, multi-wavelength, broad bandwidth frequency-domain near-infrared spectroscopy platform for real-time quantitative tissue optical imaging. Biomed. Opt. Express.

[29] Tromberg B.J., Cerussi A., Shah N., Compton M., Durkin A., Hsiang D., Butler J., Mehta R. (2005). Imaging in breast cancer: Diffuse optics in breast cancer: Detecting tumors in pre-menopausal women and monitoring neoadjuvant chemotherapy. Breast Cancer Res..

[30] Rakha E., Ellis I.O. (2007). An overview of assessment of prognostic and predictive factors in breast cancer needle core biopsy specimens. J. Clin. Pathol..

[31] Brenner R.J., Gordon L.M. (2011). Malignant seeding following percutaneous breast biopsy: Documentation with comprehensive imaging and clinical implications. Breast J..

[32] Wagih M., Komolafe A., Ullah I., Weddell A.S., Beeby S. (2023). A wearable all-printed textile-based 6.78 MHz 15 W-output wireless power transfer system and its screen-printed joule heater application. IEEE Trans. Ind. Electron..

[33] Gao S.S., Luo Q., Zhu F. (2014). Circularly Polarized Antennas.

[34] Fotopoulou K., Flynn B.W. (2010). Wireless power transfer in loosely coupled links: Coil misalignment model. IEEE Trans. Magn..

[35] Karnaushenko D.D., Karnaushenko D., Makarov D., Schmidt O.G. (2015). Compact helical antenna for smart implant applications. NPG Asia Mater..

[36] Stadler A. Radiated magnetic field of a low-frequency ferrite rod antenna. Proceedings of the 2011 7th International Conference-Workshop Compatibility and Power Electronics (CPE).

[37] Rho S., Sanders H., Morsby J.J., Smith B.D., O’Sullivan T.D. (2024). Miniature syringe-injectable wireless light source for photodynamic therapy with rose bengal. Clinical and Translational Biophotonics.

[38] Reusch D., Strydom J. (2013). Understanding the effect of PCB layout on circuit performance in a high-frequency gallium-nitride-based point of load converter. IEEE Trans. Power Electron..

[39] Sample A.P., Meyer D.T., Smith J.R. (2010). Analysis, experimental results, and range adaptation of magnetically coupled resonators for wireless power transfer. IEEE Trans. Ind. Electron..

[40] Kok H.P., Cressman E.N., Ceelen W., Brace C.L., Ivkov R., Grüll H., Ter Haar G., Wust P., Crezee J. (2020). Heating technology for malignant tumors: A review. Int. J. Hyperth..

[41] Yamagishi K., Kirino I., Takahashi I., Amano H., Takeoka S., Morimoto Y., Fujie T. (2019). Tissue-adhesive wirelessly powered optoelectronic device for metronomic photodynamic cancer therapy. Nat. Biomed. Eng..

[42] Kirino I., Fujita K., Sakanoue K., Sugita R., Yamagishi K., Takeoka S., Fujie T., Uemoto S., Morimoto Y. (2020). Metronomic photodynamic therapy using an implantable LED device and orally administered 5-aminolevulinic acid. Sci. Rep..

[43] Marquez G., Wang L.V., Lin S.-P., Schwartz J.A., Thomsen S.L. (1998). Anisotropy in the absorption and scattering spectra of chicken breast tissue. Appl. Opt..

[44] Bargo P.R., Prahl S.A., Goodell T.T., Sleven R., Koval G., Blair G., Jacques S.L. (2005). In vivo determination of optical properties of normal and tumor tissue with white light reflectance and an empirical light transport model during endoscopy. J. Biomed. Opt..

[45] Banitalebi H., Skaane P. (2005). Migration of the breast biopsy localization wire to the pulmonary hilus. Acta Radiol..

[46] Srour M.K., Kim S., Amersi F., Giuliano A.E., Chung A. (2020). Comparison of wire localization, radioactive seed, and Savi scout^®^ radar for management of surgical breast disease. Breast J..

[47] Dua S.M., Gray R.J., Keshtgar M. (2011). Strategies for localisation of impalpable breast lesions. Breast.

[48] Banys-Paluchowski M., Kühn T., Masannat Y., Rubio I., de Boniface J., Ditsch N., Karadeniz Cakmak G., Karakatsanis A., Dave R., Hahn M. (2023). Localization Techniques for Non-Palpable Breast Lesions: Current Status, Knowledge Gaps, and Rationale for the MELODY Study (EUBREAST-4/iBRA-NET, NCT 05559411). Cancers.

[49] Sharek D., Zuley M.L., Zhang J.Y., Soran A., Ahrendt G.M., Ganott M.A. (2015). Radioactive seed localization versus wire localization for lumpectomies: A comparison of outcomes. Am. J. Roentgenol..

[50] Cox C.E., Garcia-Henriquez N., Glancy M.J., Whitworth P., Cox J.M., Themar-Geck M., Prati R., Jung M., Russell S., Appleton K. (2016). Pilot study of a new nonradioactive surgical guidance technology for locating nonpalpable breast lesions. Ann. Surg. Oncol..

[51] Jeffries D.O., Dossett L.A., Jorns J.M. (2017). Localization for breast surgery: The next generation. Arch. Pathol. Lab. Med..

[52] McGugin C., Spivey T., Coopey S., Smith B., Kelly B., Gadd M., Hughes K., Dontchos B., Specht M. (2019). Radiofrequency identification tag localization is comparable to wire localization for non-palpable breast lesions. Breast Cancer Res. Treat..

[53] Patel S.N., Mango V.L., Jadeja P., Friedlander L., Desperito E., Wynn R., Feldman S., Ha R. (2018). Reflector-guided breast tumor localization versus wire localization for lumpectomies: A comparison of surgical outcomes. Clin. Imaging.

[54] Cox C.E., Russell S., Prowler V., Carter E., Beard A., Mehindru A., Blumencranz P., Allen K., Portillo M., Whitworth P. (2016). A prospective, single arm, multi-site, clinical evaluation of a nonradioactive surgical guidance technology for the location of nonpalpable breast lesions during excision. Ann. Surg. Oncol..

[55] Cha G.D., Kim D.-H., Kim D.C. (2024). Wearable and Implantable Light-Emitting Diodes and Their Biomedical Applications. Korean J. Chem. Eng..

[56] Filatova S.A., Shcherbakov I.A., Tsvetkov V.B. (2017). Optical properties of animal tissues in the wavelength range from 350 to 2600 nm. J. Biomed. Opt..

[57] Arslan H., Dolukan Y.B. (2018). Determination of the optical properties of bovine liver tissue using integrating sphere technique. Acad. Perspect..

[58] O’Sullivan T.D., Leproux A., Chen J.-H., Bahri S., Matlock A., Roblyer D., McLaren C.E., Chen W.-P., Cerussi A.E., Su M.-Y. (2013). Optical imaging correlates with magnetic resonance imaging breast density and revealscomposition changes during neoadjuvant chemotherapy. Breast Cancer Res..

