# Supplementary material for: Wirelessly Powered Visible Light-Emitting Implant for Surgical Guidance during Lumpectomy
Source: Sensors (Basel). 2024 Aug 30;24(17):5639. doi: 10.3390/s24175639 (PMC11398236; doi:10.3390/s24175639)
Supplement: Supplementary file 1 [file sensors-24-05639-s001.zip › SI description.pdf]

Wirelessly powered visible light-emitting implant for surgical guidance during lumpectomy
S. Rho et al.

Description of Supplementary Materials

Videos showing the operation of the optically-enhanced localization device in ex vivo chicken breast tissue.

a) 0.5 cm implant depth with ambient lights on
b) 0.5 cm implant depth with ambient lights off
c) 1.5 cm implant depth with ambient lights off
d) 3.0 cm implant depth with ambient lights off
